# Alpha2beta1 Integrin (VLA-2) Protects Activated Human Effector T Cells From Methotrexate-Induced Apoptosis

**DOI:** 10.3389/fimmu.2018.02269

**Published:** 2018-10-15

**Authors:** Amna Abderrazak, Mohammed-Amine El Azreq, Dalila Naci, Paul R. Fortin, Fawzi Aoudjit

**Affiliations:** ^1^Axe de Recherche sur les Maladies Infectieuses et Immunitaires, Centre de Recherche du CHU De Québec-Université Laval, Québec, QC, Canada; ^2^Division de Rhumatologie, Département de Médecine, Faculté de Médecine, Université Laval, Québec, QC, Canada; ^3^Département de Microbiologie-Infectiologie et D'immunologie, Faculté de Médecine, Université Laval, Québec, QC, Canada

**Keywords:** α2β1 integrin, collagen, cell adhesion, methotrexate, rheumatoid arthritis, apoptosis, T cells

## Abstract

β1 integrins are critical for T cell migration, survival and costimulation. The integrin α2β1, which is a receptor for collagen, also named VLA-2, is a major costimulatory pathway of effector T cells and has been implicated in arthritis pathogenesis. Herein, we have examined its ability to promote methotrexate (MTX) resistance by enhancing effector T cells survival. Our results show that attachment of anti-CD3-activated human polarized Th17 cells to collagen but not to fibronectin or laminin led to a significant reduction of MTX-induced apoptosis. The anti-CD3+collagen-rescued cells still produce significant amounts of IL-17 and IFNγ upon their reactivation indicating that their inflammatory nature is preserved. Mechanistically, we found that the prosurvival role of anti-CD3+collagen involves activation of the MTX transporter ABCC1 (ATP Binding Cassette subfamily C Member 1). Finally, the protective effect of collagen/α2β1 integrin on MTX-induced apoptosis also occurs in memory CD4^+^ T cells isolated from rheumatoid arthritis (RA) patients suggesting its clinical relevance. Together these results show that α2β1 integrin promotes MTX resistance of effector T cells, and suggest that it could contribute to the development of MTX resistance that is seen in RA.

## Introduction

Integrins are a large family of α/β transmembrane receptors playing a key role in cell-cell interactions and cell adhesion to the extracellular matrix (ECM). The very late activating antigens (VLA)-1 to -6 belonging to the β1 integrin subfamily are the most expressed ECM receptors by effector T cells. After activation, integrins induce T cell adhesion and migration through basement membranes and interstitial tissue in order to reach the inflammatory sites (1). In addition, β1 integrins also provide costimulatory signals to promote T cell survival and cytokine production (1).

The collagen-binding integrin α2β1 (VLA-2) has recently gained significant attention as one of the major integrin involved in T cell-mediated immunity. It is expressed on effector/memory T cells found in inflammatory tissues but not on naïve T cells (2–4). α2β1 integrin is found on Th1 cells (5) and it is the major integrin that binds collagen, which is expressed by human Th17 cells (6).

α2β1 integrin has been involved in Th17 and Th1 cell adhesion to collagen and in costimulation of IL-17 and IFNγ production (5–7). Furthermore, it promotes the survival of human effector T cells by inhibiting Fas-induced apoptosis (8). *In vivo* studies showed the implication of α2β1 integrin in the development of inflammatory diseases including experimental colitis (9), experimental autoimmune encephalomyelitis (10) and arthritis. In this case, we have shown that α2β1 integrin is expressed on RA synovial Th17 cells and its blockade reduces severity of collagen-induced arthritis and IL-7-induced bone loss in mice by reducing Th17 cell numbers and activity in the synovial tissue (11, 12).

RA is a disabling disease in which Th17 and Th1 cells play a central role in the resulting synovitis and cartilage and bone erosion. Despite the introduction of several biologics, MTX is still the first line in RA therapy and the most frequently used disease-modifying anti-rheumatic drug. However, 30–40% of patients fail to respond or end-up developing resistance, thus becoming unresponsive (13, 14). The mechanisms accounting for MTX resistance in RA are still unclear although increased metabolism, altered target enzymes, and defective cellular uptake or increased MTX efflux through the expression and activity of ATP-binding cassette (ABC) drug transporters have been proposed (13, 14). These drug transporters, which are involved in cancer chemoresistance (15), have the ability to function, in an ATP-dependent manner, as a pump in order to extrude various endogenous (steroids, metabolites, ions) or exogenous substrates (drugs) out of the cells.

MTX can act by blocking cell proliferation and cytokine production (16). However, one major effect of MTX is the induction of apoptosis in proliferating activated/effector T cells (16, 17). Decreased T cell numbers in the synovium of RA patients treated with MTX has also been reported (18, 19). Thus, it is likely that factors that promote resistance of effector T cells to apoptosis may have a significant role in MTX resistance. Since α2β1 integrin plays an important role in the survival and costimulation of effector T cell and in arthritis pathogenesis, we tested its contribution to MTX resistance using a tailored *in vitro* T cell model and T cells from RA patients. Our results show that α2β1 protects activated human polarized Th17 cells and RA effector/memory T cells from MTX-induced apoptosis through the ABC drug transporter ABCC1. Taken together our findings indicate that α2β1 integrin promotes Th17 cell resistance to MTX, and thus it could contribute to MTX resistance that is observed in RA.

## Materials and methods

### Reagents and antibodies

Cell culture medium, X-vivo 15, was purchased from Lonza technologies (Walkersville, MD). Human cytokines (IL-6, TGF-β, IL-2, IL-1β, and IL-23) were purchased from R&D Systems (Minneapolis, MN). Type II collagen (referred hereafter as collagen) was from EPC Elastin Products Company (Owensville, MO), fibronectin, was from Sigma-Millipore (St. Louis, MO) and laminin-8 was from Biolamina (Stockholm, Sweden). The ABCC1 inhibitor MK571 and calcein-AM were from Calbiochem (San Diego, CA). The ABCG2 inhibitor, fumitremorgin c and ABCC1 inhibitor, reversan were from Sigma-Millipore (St-Louis, MO). MTX, the blocking anti-human α2 integrin (P1E6), the blocking anti-α2β1 integrin (BHA2.1) and their appropriate isotypic control antibodies were from EMD Millipore (Billerica, MA). The blocking anti-human β1 integrin (4B4) and its control isotypic antibody were purchased from Beckman Coulter (Brea, CA). CD3/CD28 Dynabeads were from Invitrogen Dynal AS (Oslo, Norway). The anti-CD3 mAb (OKT3), PE-conjugated anti-human IFNγ (B27), PE-conjugated anti-human α2 integrin (12F1), FITC-conjugated anti-human ABCC1 (QCRL-3), Alexa 647-conjugated anti-human IL-17 (N49-653), PE-conjugated anti-ABCG2 (ATP-binding cassette sub-family G member 2) (5D3), their appropriate control isotypic antibodies and the FITC-annexin V apoptotic kit were from BD Biosciences (San Diego, USA). Anti-β-actin (C2) and anti-caspase-3 (E-8) antibodies were from Santa Cruz Biotechnology (Santa Cruz, CA).

### Ethical statement

Our study was approved by the CHU de Québec-Université Laval ethical committee for clinical research. Healthy adult blood donors were recruited through the clinical research facility at the CHU de Québec-Université Laval Research Center. RA patients were recruited through the CHU de Québec-Université Laval Systemic Autoimmune Rheumatic Diseases Biobank and Repository Database. The CHU de Québec-Université Laval ethics committee has approved the biobank and its management (CÉR #B13-06-1243). Our experiments were conducted in accordance with the local ethical guidelines and regulations. RA patients and healthy donors provided and signed written consent forms before blood collection.

### Naïve CD4^+^ T cells isolation and Th17 differentiation

PBMCs were collected from blood of healthy subject volunteers using ficoll gradients. Next we purified primary human naïve CD4^+^T cells by negative selection using the EasySep human Naïve CD4^+^ Cell Enrichment Kit from StemCell Technologies (Vancouver, BC) as described in manufacturer's instructions. We then generated Th17 cells after activation of naïve CD4^+^ T cells during 6 days with CD3/CD28 beads (one bead/cell) in serum-free X-vivo medium in the presence of Th17 polarizing cytokines (TGFβ, IL-1β, IL-6, and IL-23) as we previously described (12).

### RA patients and CD45RO^+^ memory T cells isolation

PBMCs were isolated from peripheral blood of RA patients (*n* = 5) with established disease according to the American College of Rheumatology criteria (20). The patients were between 37 and 71 y old with disease duration of one year and received prednisone and MTX. We purified memory CD4^+^ T cells by negative selection using an appropriate kit from StemCell Technologies (Vancouver, BC). CD4^+^CD45RO^+^ T cells (1 × 10^6^) in 1 ml of X-vivo medium were expanded with CD3/CD28 beads (one bead/cell) and IL-2 (50 Units/ml) for 6 days.

### T cell activation and apoptosis

Wells were first coated with 2 μg/ml of anti-CD3 mAb for 24 h at 4°C, after which, they were washed with PBS. The wells with or without anti-CD3 were then coated with 2 μg/ml collagen for 3 h at 37°C. After three washes with PBS, we seeded the cells for 3 h, and treated them with 10 μM of MTX for 24 h at 37°C. Cell apoptosis was determined using annexin V-FITC staining and measured by flow cytometry (BD FACSCALIBUR II cytometer).

### Cell adhesion assays

Cell adhesion assays were conducted as described (7). A 96-well microtiter plate (TC plate, flat bottom, Falcon) was coated with 20 μg/ml of matrix proteins diluted in PBS overnight at 4°C. After three washes with PBS, wells were blocked with 1% BSA for 1 h at 37°C. Then, 10^5^ cells in 100 μl of X-vivo medium activated or not with anti-CD3-coated beads were seeded into the wells. After 1 h, the cells were washed with PBS and fixed in a PBS solution containing 1% formaldehyde for 1 h at room temperature. The cells were washed and stained with a methanol solution containing 0.5% crystal violet. After four washes, we lysed the cells using a 1% SDS solution and determined the absorbance at 600 nm using an ELISA plate reader.

### Expression of α2 integrin, ABCC1, and ABCG2 transporters

The cells were stained with (PE-conjugated) isotypic control, anti-α2 integrin, and anti-ABCG2 antibodies for 30 min on ice. For ABCC1 expression, we first permeabilized and fixed the cells with a cytoFix/CytoPerm kit (BD Biosciences) for 20 min, after which, we incubated them with FITC-conjugated isotypic control or anti-ABCC1 antibodies. The cells were washed and then analyzed for α2 integrin, ABCC1, and ABCG2 expression levels by flow cytometry (BD FACSCalibur II cytometer).

### Quantitative RT-PCR

Total RNA isolation and cDNA preparation were carried out as we previously described (6). PCR reactions were performed with 1 μl of cDNA in a 20 μl of total volume containing SYBR Green I (Invitrogen, CA) for 35 cycles in Rotor-Gene 3000 operated with Rotor-Gene Software 9 version 6.0.19 (Corbett Research, Mortlake, New South Wales, Australia). For specificity, we performed the Melts procedure as we previously described (6). The housekeeping gene β*-actin* was used for normalization of *ABCC1* and *ABCG2* gene expression. As a calibrator, control PCR reactions using RNA from MCF-7 cells, which express both ABCC1 and ABCG2 (21) were used. Relative gene expression was calculated using the ΔΔCt comparative method and the formula: ΔΔCt = (Ct target gene-Ct β-actin) sample–(Ct target gene–Ct β-actin) calibrator. Primers for human *ABCC1, ABCG2*, and β*-actin* genes were as previously described (6, 22).

### Caspase-3 activation and western blot analysis

Caspase-3 activation was evaluated by Western blot using anti-caspase-3 antibody, which recognizes the native and active caspase-3 fragments, as we previously described (23). Blots were stripped and then reprobed for loading control with anti-β-actin antibody.

### IL-17 and IFNγ quantification

After MTX treatment in the presence of anti-CD3+collagen, viable polarized Th17 cells (rescued cells) were isolated through a ficoll gradient (viability of more than 95%). Then, equal cell numbers of rescued cells and control cells (non-treated) were restimulated with anti-CD3+collagen for 24 h. The supernatants were collected for ELISA analysis in order to quantify IL-17 and IFNγ production using ELISA kits from R&D Systems (Minneapolis, USA).

For intracellular cytokine staining, arthritic effector/memory CD4^+^ T cells were activated with 50 ng/ml of Phorbol-12-Myristate 13-Acetate (PMA) and 750 ng/ml of ionomycin in the presence of BD Golgi Plug (2 μg/ml) for 4 h. After permeabilisation and fixation with cytoFix/CytoPerm kit (BD Biosciences), the cells were stained with intracellular anti-IL-17 and anti-IFNγ antibodies and positive cells were evaluated by FACS analysis (BD FACSCalibur II).

### Calcein-AM intracellular content

Human polarized Th17 cells in X-vivo medium were activated for 3 h with anti-CD3+collagen. The cells were then cultured for 1 h in medium containing 1 nM of calcein-AM, after which the cells were washed. The intracellular levels of calcein-AM were determined by flow cytometry (BD FACSCalibur II) using the FL-1 channel. Results are expressed as percentage of positive cells times (x) mean fluorescence intensity (MFI) giving a metric referred as integrated MFI, which reflects the total expression in positive cells (24). This method of quantification has been used in various studies including ours (25–28) and has therefore been applied throughout this study.

### Statistical analysis

The Student's *t*-test was used and *p*<0.05 was considered significant.

## Results

### Collagen protects human polarized Th17 cells from MTX-induced apoptosis

**Figure 1:**
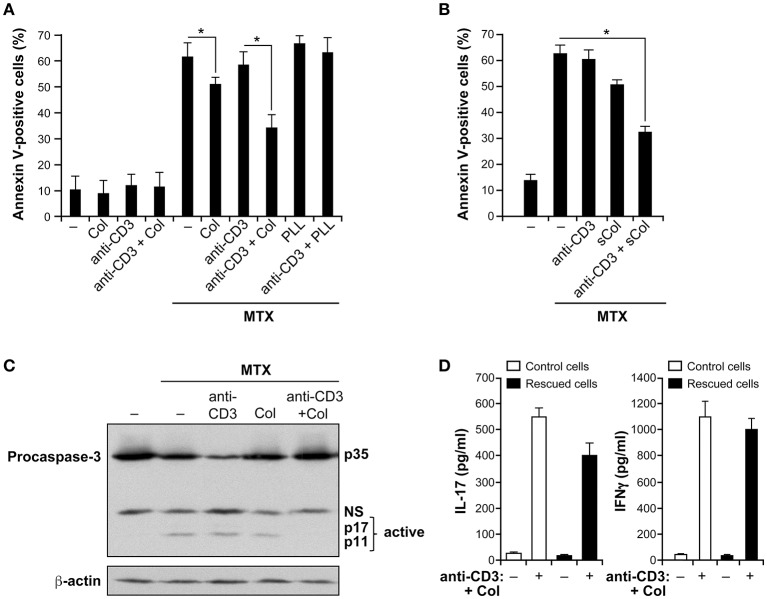
Collagen promotes MTX resistance of activated human polarized Th17 cells. **(A)** Collagen protects human polarized Th17 cells from MTX-induced apoptosis. Cells were incubated or not (−) for 3 h with coated anti-CD3 mAb, collagen (Col) or as a control with poly-L-lysine (PLL) or in combination as described. Then, they were treated for 24 h with 10 μM of methotrexate (MTX) and apoptosis was evaluated using FITC-conjugated annexin V staining and FACS analysis. **(B)** Protective effect of soluble collagen. Cells were activated or not (–) for 3 h with 50 μg/ml of soluble Col (sCol), anti-CD3 mAb and with a combination of anti-CD3+sCol. They were then treated for 24 h with MTX and apoptosis was evaluated by annexin V staining and FACS analysis. **(C)** Collagen inhibits MTX-induced caspase-3 activation. Cells were activated for 3 h as indicated and then treated for 12 h with MTX. Cell lysates were prepared and analyzed for caspase-3 activation by Western blot (NS, non-specific band). Blots were stripped and reprobed with anti-β-actin Ab as a loading control. The results are representative of three independent experiments. **(D)** Anti-CD3+collagen stimulation preserves the inflammatory nature of polarized Th17 cells. Cells were activated with anti-CD3+Col and then treated with MTX. After 24 h, viable cells were isolated and stimulated or not (−) along with control cells with anti-CD3+Col. Supernatants were harvested 24 h later and the production of IL-17 and IFNγ was quantified by ELISA. The results in panels **(A,B,D)** are presented as mean ± SD calculated from three to five different experiments conducted in triplicates with human T cells isolated from different healthy blood donors. ^*^p < 0.05.

In order to determine whether α2β1 integrin provides a survival advantage to Th17 cells, we first studied the effect of collagen on MTX-induced apoptosis in human polarized Th17 cells. As previously shown, 15–20 % of the cells produce IL-17 (12) (Figure S1 in Supplementary material). MTX has been shown to induce apoptosis at 0.1–10 μM, which mimic the low doses administered to RA patients (17, 29, 30) and we have found that MTX at 10 μM is the optimal dose in our cell model. As shown in Figure 1A, adhesion to collagen slightly reduces MTX-induced apoptosis of polarized Th17 cells as evidenced by the decrease in annexin-positive cells compared with cells treated only with MTX. Anti-CD3 alone did not show any effect on cell survival. However, culture of the cells on anti-CD3+collagen led to a 40% reduction in apoptosis. As a control, the non-integrin ligand poly-L-lysine had no effect on MTX-induced apoptosis. Of note, we also found that anti-CD3+collagen reduces MTX-induced apoptosis by 35–40% when MTX was used at 0.1 and 1 μM (data not shown).

To determine if the collagen effect is due to increased cell contacts with coated anti-CD3 mAb as a result of cell adhesion, we tested if collagen still has the ability to protect polarized Th17 cells from MTX-induced apoptosis when added in soluble form. We found that the soluble form of collagen has the same ability as immobilized collagen to protect the cells from MTX-induced apoptosis (Figure 1B) suggesting that the pro-survival effect of collagen is a result of its signaling function. Together, these results indicate that although collagen by itself has a small effect, it is the combination of anti-CD3+collagen that has produced the most important effect on cell survival.

Because MTX-induced apoptosis is associated with caspase-3 activation, we determined if anti-CD3+collagen costimulation inhibited caspase-3 activation in MTX-treated cells. Treatment of polarized Th17 cells with MTX is associated with caspase-3 activation as determined by the proteolysis of procaspase-3 leading to the generation of caspase-3 active fragments (Figure 1C). Activation of the cells with anti-CD3 mAb had no effect whereas their activation with collagen alone had only a minor effect. However, activation with anti-CD3+collagen effectively inhibited MTX-induced caspase-3 activation further indicating that anti-CD3+collagen signaling protects polarized Th17 cells from MTX-induced apoptosis.

To determine if the anti-CD3+collagen-rescued cells from MTX treatment were still able to produce IL-17, rescued cells (viable cells) were isolated through a ficoll gradient and restimulated with anti-CD3+collagen. The results show that these cells still produce significant amounts of IL-17 when compared to control cells that have not been treated with MTX (Figure 1D). In addition, since human polarized Th17 cells also contain Th1 cells (31, 32), we examined the production of IFNγ and found that upon restimulation with anti-CD3+collagen, MTX-rescued cells produce comparable levels of IFNγ than control cells (Figure 1D). These results indicate that under MTX conditions, costimulation with anti-CD3+collagen preserves the inflammatory nature of human polarized Th17 cells. As a control, costimulation of the rescued cells with anti-CD3+anti-CD28 antibodies also led to the production of comparable levels of IL-17 and IFNγ than control cells (data not shown) further supporting that the rescued cells kept their inflammatory function.

### The pro-survival role of collagen is mediated via α2β1 integrin

**Figure 2:**
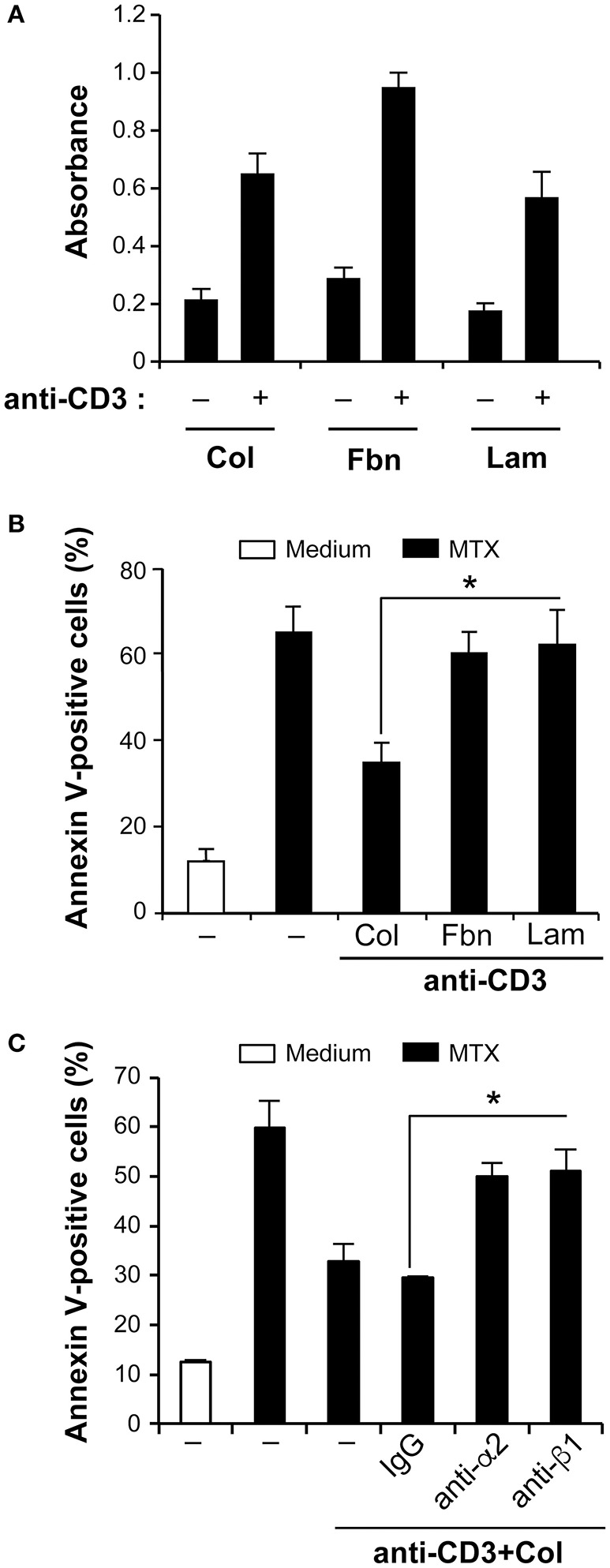
Collagen-α2β1 integrin interaction protects human polarized Th17 cells from MTX-induced apoptosis. **(A)** Human polarized Th17 cells adhesion to matrix proteins. Control and anti-CD3-activated cells were cultured for 1 h in wells coated with Col, fibronectin (Fbn) or laminin (Lam). Cell Adhesion was quantified as described in the Materials and Methods section. **(B)** Differential effect of Col on MTX-induced apoptosis. Human polarized Th17 cells were cultured for 3 h with Col, Fbn or Lam in the presence of anti-CD3 mAb after which they were treated for 24 h with MTX. The cells were stained with annexin V and apoptosis was determined by FACS analysis. **(C)** Cells were first incubated for 1 h with 10 μg/ml of anti-alpha2 integrin (clone P1E6) and anti-beta1 integrin (clone 4B4) blocking mAbs or with isotypic control IgG. They were then activated with anti-CD3+Col and treated with MTX for 24 h. The cells were stained with annexin V and apoptosis was determined by FACS analysis. Results are presented as mean ± SD calculated from three independent experiments conducted in triplicates with T cells obtained from three different healthy donors. ^*^p < 0.05.

Effector T cells are characterized by the expression of several members of β1 integrins that play an important role in their interactions with ECM proteins (1–4). Besides collagen, fibronectin and laminin are also two major matrix proteins. We thus examined if fibronectin and laminin, which bind to α4β1/α5β1 and α3β1/α6β1 integrins respectively could have a similar protective effect as collagen. To this end, we first assessed the ability of human polarized Th17 cells to attach to the various matrices. The results show that human polarized Th17 cells are capable of adhering to all matrices. Cell adhesion to fibronectin is slightly higher than to the other two matrices upon anti-CD3 activation, which increases cell adhesion to all matrices (Figure 2A). However, fibronectin and laminin had no effect on MTX-induced apoptosis in anti-CD3-treated cells (Figure 2B). Similar findings were obtained when fibronectin and laminin were added in their soluble forms (data not shown). Because human polarized Th17 cells express and use α2β1 integrin to attach to collagen, we verified if it transduces the protective effect of collagen. We found that incubation of the cells with blocking anti-β1 and anti-α2 integrin antibodies abrogated the collagen protective effect on MTX-induced apoptosis whereas the control antibody had no effect (Figure 2C). These results suggest the selective implication of α2β1 integrin in the resistance of effector T cells to MTX.

### Collagen promotes MTX resistance via the ABCC1 drug transporter

**Figure 3:**
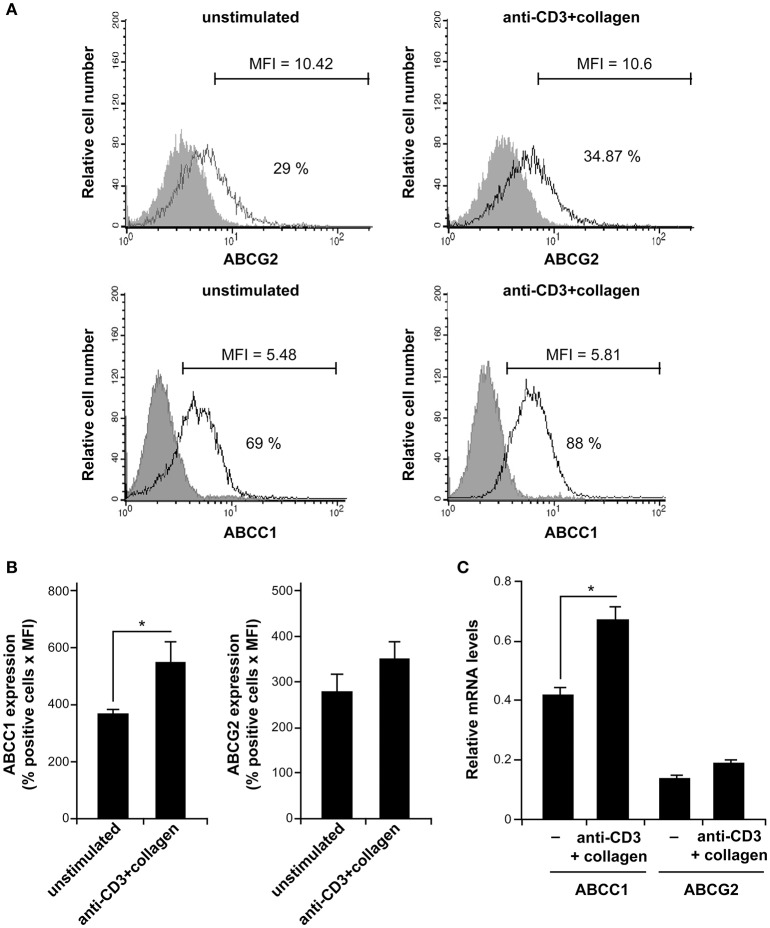
Human polarized Th17 cells express ABCC1. **(A)** ABCC1 and ABCG2 expression. After 12 h of culture with or without stimulation with anti-CD3+Col, the cells were stained with specific anti-ABCC1 and anti-ABCG2 antibodies and analyzed by FACS analysis. The gray areas represent control stainings with appropriate conjugated-isotypic antibodies. Results are representative of five independent experiments conducted with T cells obtained from five different donors. The % of positive cells and MFI are indicated in each histogram. **(B)** Quantification of ABCC1 and ABCG2 expression levels. Results are expressed as % positive cells x MFI and the values represent mean ± SD calculated from five independent experiments conducted with T cells obtained from five different donors. **(C)** The ABCC1 and ABCG2 mRNA levels expressed in human polarized Th17 cells at the basal level and after anti-CD3+collagen stimulation were determined by qRT-PCR. The results represent mean values ± SD calculated from three independent experiments conducted in triplicates with T cells obtained from three different donors. ^*^p < 0.05.

We then sought to determine the mechanisms by which T cell adhesion to collagen promotes MTX resistance in activated human polarized Th17 cells. The drug transporters of the ABC subfamily have been highly associated with chemoresistance. Accordingly, we examined whether they could be involved in our cell model. ABCC1 and ABCG2 also known as MRP-1 and BCRP1 respectively are two major MTX transporters and reported to be expressed in T cells (33, 34). Thus, we first assessed whether these two transporters were expressed in human polarized Th17 cells. The results show that polarized human Th17 cells express slightly higher levels of ABCC1 than ABCG2 especially upon anti-CD3+collagen costimulation (Figure 3A). Quantification analysis indicates a 1.5 × fold increase in ABCC1 levels upon anti-CD3+collagen costimulation, which had no significant effect on ABCG2 protein levels (Figure 3B). The ABCC1 levels are 1.57 × fold higher than ABCG2 levels in anti-CD3+collagen-costimulated cells.

Because of the low levels detected by FACS, we sought to analyse the expression levels of ABCC1 and ABCG2 by quantitative RT-PCR (qRT-PCR), which is the preferred method for studying expression of ABC transporters in immune cells. Indeed, although functional but detection of protein levels of ABC transporters in human T cells is weak (35–37). We found that ABCC1 mRNA levels are three times higher than those of ABCG2 and costimulation with anti-CD3+collagen increases ABCC1 mRNA levels by 1.6 × fold but had no effect on ABCG2 mRNA levels (Figure 3C). Together, these results indicate that human polarized Th17 cells express higher levels of ABCC1 than ABCG2, which is in agreement with recent findings in human Th1 and Th17 cells (37). The discrepancies between the FACS and qRT-PCR quantification data with regard to the relative levels of ABCC1 *vs*. ABCG2 could be due to the low levels detected by FACS, which could be due to the efficacy of antibodies, and to the differences in the staining methods for ABCC1 and ABCG2.

**Figure 4:**
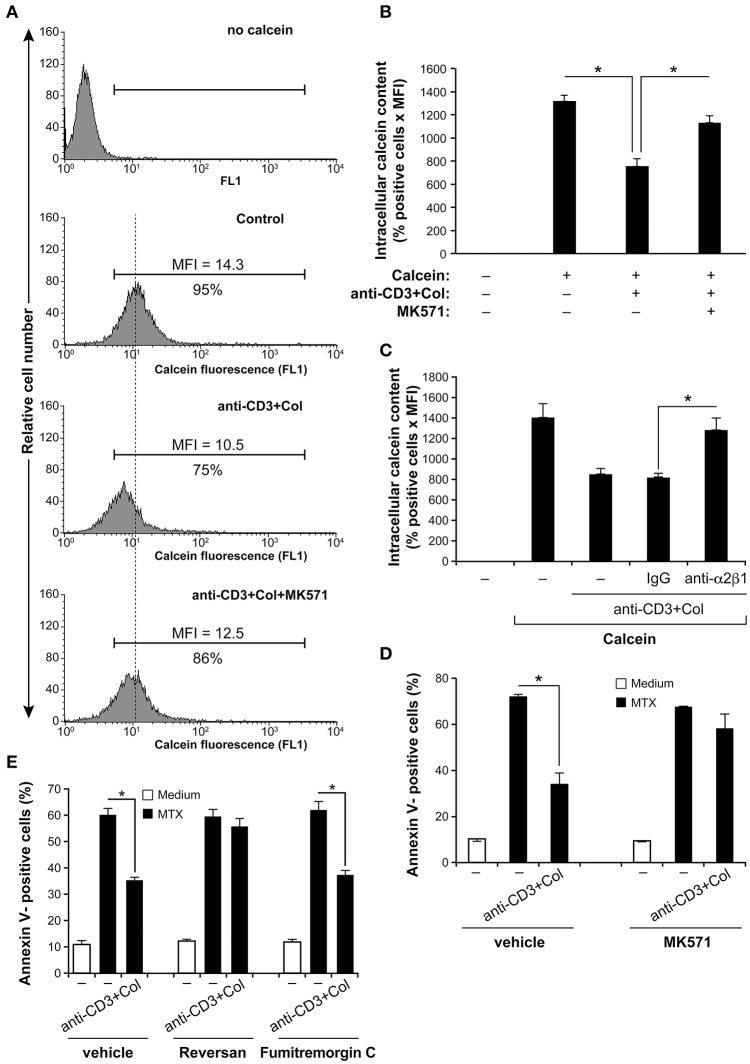
Collagen promotes MTX resistance of activated human polarized Th17 cells *via* the drug transporter ABCC1. **(A)** Anti-CD3+Col costimulation reduces ABCC1 activity. After 3 h activation with anti-CD3+Col, the cells were incubated for 1 h with 1 nM of calcein-AM. As indicated, cells were pre-treated with 10 μM of MK571 (ABCC1 inhibitor) before being stimulated with anti-CD3+Col. Intracellular calcein-AM content was then determined by FACS using the FL-1 channel. Representative FACS profiles of intracellular calcein-AM content are shown. The % of positive cells and MFI are indicated. **(B)** Quantification of intracellular calcein-AM content. **(C)** The blocking anti-α2β1 integrin (BHA2.1) abolishes the effect of anti-CD3+Col on calcein-AM efflux. Cells were first incubated or not for 1 h with 10 μg/ml of BHA2.1 mAb or with isotypic control IgG, activated or not with anti-CD3+Col and then loaded with calcein-AM. Intracellular calcein-AM content was then determined by FACS analysis and quantified as above. **(D)** The MK571 inhibitor abolished the protective effect of anti-CD3+Col on MTX-induced apoptosis. Cells were treated as indicated and after 24 h of MTX treatment, the cells were stained with annexin V and analyzed by FACS. **(E)** The ABCC1 inhibitor reversan (10 μM) but not the ABCG2 inhibitor fumitremorgin C (10 μM) abrogates the protective effect of anti-CD3+collagen on MTX-induced apoptosis. Results (**(B–E)** panels) are presented as mean ± SD calculated from three independent experiments performed in triplicates with T cells obtained from three different donors. ^*^p < 0.05.

We then determined the functionality of ABCC1 by evaluating the content of intracellular calcein-AM by FACS analysis. Calcein-AM is a specific substrate for ABCC1 and has been used as a surrogate for MTX efflux and ABCC1 activity in multiple studies (38, 39). As shown, activation of the cells with anti-CD3+collagen reduces calcein-AM intracellular content by reducing the % of positive cells as well as the MFI, and this regulatory effect is partially reversed by the specific ABCC1 inhibitor, MK571 (Figure 4A). Quantification analysis taking into account the number of positive cells and MFI indicates a 40% reduction in total intracellular calcein-AM levels upon activation with anti-CD3+collagen and the MK571 inhibitor blocked the effect of anti-CD3+collagen by about 75% (Figure 4B). As a control, we also found that the effect of collagen on calcein-AM efflux is abolished with a blocking anti-α2β1 mAb (Figure 4C). These results indicate that collagen via α2β1 integrin enhances the expression and the function of ABCC1 in anti-CD3-activated polarized Th17 cells.

Using the specific ABCC1 inhibitor, MK571, we then studied the role of ABCC1 in MTX resistance of human polarized Th17 cells. Treatment with the MK571 inhibitor did not show any effect on MTX-induced apoptosis but inhibited the anti-CD3+collagen protective effect (Figure 4D). To confirm the effect of the MK571 inhibitor, we evaluated the effect of a second ABCC1 inhibitor named reversan (40). Similar to MK571, reversan abolished the protective effect of anti-CD3+collagen (Figure 4E). Interestingly, the specific ABCG2 inhibitor fumitremorgin C (41) had no effect. Taken together, these results show that collagen promotes MTX resistance of anti-CD3-activated human polarized Th17 cells by upregulating the function of ABCC1.

### Collagen promotes MTX resistance of arthritic T cells

**Figure 5:**
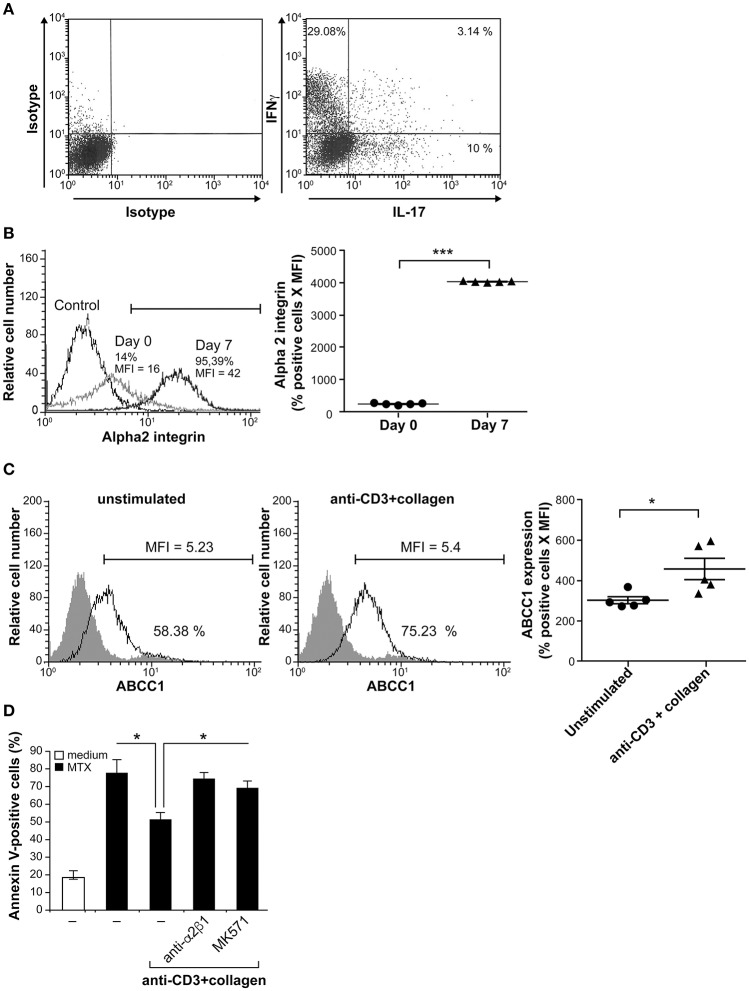
Collagen protects arthritic effector/memory CD4^+^ T cells from MTX-induced apoptosis. **(A)** FACS analysis of Th1 and Th17 in arthritic T cells. CD4^+^CD45RO^+^ T cells collected from peripheral blood of RA patients contain IL-17- and IFNγ-producing cells. After activation with PMA and ionomycin for 4 h in the presence of BD Golgi plug, the cells were stained with intracellular anti-IL-17 and anti-IFNγ antibodies and analyzed by FACS. The results are representative of five different RA patients. **(B)** α2 integrin expression was evaluated by FACS analysis on day 0 and day 7 (one week after *in vitro* expansion). **(C)** ABCC1 expression was evaluated by FACS analysis on day 7. The gray areas represent staining with appropriate conjugated-isotypic control antibodies. The scatter-bar plots (right panels in **(B)** and **(C)**) represent quantification of α2 integrin and ABCC1 expression levels respectively. Results are presented as mean ± SEM calculated from five independent experiments conducted with five different RA patients. ^***^p < 0.001; ^*^p < 0.05. **(D)** The blocking anti-α2β1 integrin mAb and the ABCC1 inhibitor MK571 inhibit the protective effect of anti-CD3+Col costimulation. *In vitro* expanded CD4^+^CD45RO^+^ T cells were stimulated during 3 h with anti-CD3^+^Col, followed by a 24 h treatment with MTX. As indicated, the cells were we first pre-treated for 1 h prior their activation, with the blocking anti-α2β1 mAb (BHA2.1) and with the MK571 inhibitor. Apoptosis was measured following annexin V staining and FACS analysis. Results are presented as mean ± SD calculated from three different experiments conducted in triplicates with CD4^+^CD45RO^+^ T cells isolated from three RA patients. ^*^p < 0.05.

Next we analyzed whether the pro-survival role of collagen/α2β1 interaction occurs in RA with effector CD4^+^T cells that have not been polarized *in vitro*. To this end, we examined whether it promotes MTX resistance of CD4^+^ memory T cells isolated from RA patients. In agreement, we found that RA peripheral blood CD4^+^CD45RO^+^ T cells contain Th1, Th17, and Th17/Th1 subsets (Figure 5A); the main pathogenic Th cells involved in RA pathogenesis (42). Because these cells are in a quiescent state, we reactivated them with anti-CD3 and anti-CD28 mAbs and expanded them with IL-2 for 1 week to sensitize them to MTX-induced apoptosis. Their activation also increased the levels of α2 integrin (Figure 5B) consistent with the upregulation of α2 integrin upon T cell activation and effector differentiation (1–4, 43). Furthermore, we found that these cells also express the drug transporter ABCC1 and their activation with anti-CD3+collagen also increases ABCC1 levels (Figure 5C).

We then examined the response of arthritic effector T cells to MTX. Activation of expanded-RA peripheral blood CD4^+^CD45RO^+^ T cells with anti-CD3+collagen reduced MTX-induced apoptosis by 38%. This effect was mediated by α2β1 integrin since it was reversed by incubating the cells with the blocking anti-α2β1 mAb (Figure 5D). In addition, the ABCC1 inhibitor MK571 also abrogated the pro-survival effect of collagen in anti-CD3-activated arthritic T cells (Figure 5D). Together these results show that arthritic effector T cells are also protected from MTX-induced apoptosis upon their attachment to collagen via α2β1/ABCC1 pathway.

## Discussion

The mechanisms underlying MTX failure in RA are still unclear and their elucidation could lead to more efficient therapies. Th17 and Th1 cells are critical effector cells in joint tissue damage and their resistance to MTX could contribute to MTX therapy failure in RA. Since MTX is associated with apoptosis of effector T cells, we have considered whether β1 integrins, which play an important role in cell survival, would provide a survival advantage to effector T cells under MTX therapeutic setting.

We found that collagen, which is abundant in the synovium, *via* its receptor, the integrin α2β1, protects human polarized Th17 cells from MTX-induced apoptosis. The strongest effect of collagen is seen only in anti-CD3-activated cells. This is likely due to the increased attachment of the cells to collagen upon stimulation via the T cell receptor (TCR), which occurs through α2β1 (Figure 2A) (6). Although TCR/CD3 activation alone did not protect human polarized Th17 from MTX-induced apoptosis, it is not excluded that additional pathways activated by the TCR/CD3 engagement could cooperate with α2β1 integrin in mediating T cell survival. Anti-CD3-activated human polarized Th17 cells also attach to fibronectin and laminin but these matrices did not influence MTX-induced apoptosis suggesting a differential implication of β1 integrins in the modulation of MTX-induced apoptosis, which could be due to their differential signaling capacities since both α and β integrin chains are known to induce cell signaling. The role of α2β1 integrin in T cell survival is reminiscent to previous work that showed that this collagen-binding integrin inhibits Fas-induced apoptosis in human effector T cells whereas fibronectin- and laminin-binding integrins did not (8). Taken together, these studies indicate that in addition to Fas-induced apoptosis, α2β1 integrin has also the ability to protect effector T cells during therapeutic setting.

MTX has been shown to act by blocking cell proliferation and cytokine production (16). Our results indicate that anti-CD3+collagen-rescued cells have kept their ability to produce IL-17 and IFNγ upon restimulation. This suggests that in addition of promoting survival, the anti-CD3+collagen signaling pathway also kept the inflammatory nature of human polarized Th17 cells, thus supporting the role of α2β1 integrin in MTX resistance.

We have also shown that the pro-survival role of α2β1 integrin under MTX setting occurs in the context of RA. Despite the fact that we did not have access to synovial fluids and were limited in RA samples and T cell numbers, we showed that memory CD4^+^ T cells isolated from peripheral blood of RA patients are also rescued from MTX-induced apoptosis suggesting that this mechanism could be clinically relevant. Although, we could not examine the role of α2β1 integrin specifically in RA Th17 *vs*. Th1 cells, it is likely that a similar mechanism occurs in both populations since α2β1 integrin is a costimulatory molecule for both Th1 and Th17 (5–7), and as shown in Figure 1D, both Th1 and Th17 can be protected by anti-CD3+collagen costimulation since MTX-rescued cells are able to produce IL-17 and IFNγ to the same extent as control cells upon restimulation. α2β1 integrin promotes arthritis pathogenesis by acting on synovial Th17 cells (11, 12) and on synovial fibroblasts (44). Whether it promotes MTX resistance of other cell types involved in arthritis pathogenesis remains to be determined.

Cell adhesion-mediated drug resistance is a well-studied process in oncology. Indeed, several studies both in solid tumors and in hematological malignancies have shown that attachment to ECM *via* integrins promotes the resistance of cancer cells to multiple drugs used in chemotherapy (45, 46). In this context, α2β1 integrin, which is an important integrin in cancer, has also been associated with drug resistance (47). This is particularly the case in human acute T lymphoblastic leukemia (T-ALL). Attachment of human T-ALL cell lines and blasts to collagen protected them from the cytotoxic action of doxorubicin (25, 48). The study reported herein indicates that the role of α2β1 integrin in chemoresistance is conserved in normal T cells and autoimmune diseases and suggests that as in cancer, cell adhesion-mediated drug resistance could contribute to therapy failures that are observed in the treatment of autoimmune diseases.

Mechanistically, our results support the implication of the drug transporter ABCC1 in MTX resistance. ABCG2 and ABCC1 are two major MTX transporters that have been associated with T cells (33, 34). We found that human effector T cells express higher levels of ABCC1 than ABCG2 and activation with anti-CD3+collagen enhanced the efflux activity of ABCC1. Furthermore, the specific ABCC1 inhibitors, MK571 and reversan, inhibited the protective effect of anti-CD3+collagen both in human polarized Th17 cells as well as in RA effector/memory T cells, whereas the ABCG2 inhibitor (fumitremorgin C) had no effect. A recent study found that CD147 promotes MTX resistance of immune cells through ABCG2 in psoriasis (49). However, this was analyzed in PBMCs and not specifically in T cells. ABCG2 has also been associated with MTX resistance of macrophages in RA synovial tissue but it was not detected in synovial T cells, which were rather shown to express ABCC1 (50). Recently, it was found that a subset of human inflammatory Th17 cells (Th17.1) express the drug transporter ABCB1 (also named PgP) and were refractory to glucocorticoids, although this occurred independently from ABCB1 (37). The authors of this study also found that ABCC1 is expressed at higher levels than ABCB1 and ABCG2 in the different human effector T cells including Th1, Th2, Th17, and the inflammatory Th17.1 subset. Together these studies suggest that ABCC1 could be the most important MTX transporter in T cells, whereas ABCG2 could be associated with monocytes/macrophages.

ABCC1, ABCG2 as well as ABCB1 are all expressed in RA (33, 51–53) but a clear implication of a particular transporter in predicting MTX response is unclear. However, the majority of these studies focused on RA PBMCs, which could be phenotypically and functionally different from the synovial immune cells that are directly associated with tissue damage. Indeed, microenvironmental cues are likely to influence the expression and function of MTX transporters expressed by the various immune cells infiltrating the synovium. In this context, our study reveals that integrins and attachment to collagen, which is abundant in the synovium, could be important factors in regulating activity of MTX transporters. The mechanisms of MTX resistance in RA are complex and could involve additional factors. Along these lines, a recent study showed that MTX resistance in RA could be correlated with weak levels of CD39 expressed by regulatory T cells (54).

Our results showed that activation with anti-CD3+collagen increased only slightly the ABCC1 levels suggesting that the effect of anti-CD3+collagen occurs mainly at the level of ABCC1 activity. This is in agreement with studies reporting that chemotaxis and migration of Langerhans cells and T cells to the lymph nodes is associated with the activation of ABCC1, which mediates efflux of sphingolipid and cysteinyl leukotriene (55, 56). Similarly, antigen-induced export of sphingosine-1 phosphate from mast cells is dependent on ABCC1 activation (57). It has previously been shown that ABCC1 localization in membrane lipid rafts and actin polymerization are both necessary for its activity (58). In addition, collagen/α2β1 integrin signaling enhances ABCC1 activity and doxorubicin resistance in acute T lymphoblastic leukemia *via* actin polymerization (25). Although the role of lipid rafts in ABCC1 activation is not firmly established (59), α2β1 integrin has previously been shown to localize into lipid rafts (60, 61). Thus, it is possible that anti-CD3-induced attachment of human effector T cells to collagen leads to cytoskeletal reorganization and thereby to increased ABCC1 localization, stability and function. Nonetheless, additional mechanisms such as receptor recycling and increased ATP production could also be involved.

Although reduction of purine-pyrimidine levels has been associated with MTX-induced apoptosis in T cells (16, 17, 29) folate co-therapy did not interfere with MTX clinical benefit (62) suggesting that apoptosis may not be critical for MTX action. However, this cannot be excluded since recent studies found that MTX-induced apoptosis in T cells is dependent on a reduction in tetrahydrobiopterin but not purine-pyrimidine levels (63, 64). Clearly additional studies are required to understand how MTX works in RA but regardless of its mode of action, the MTX resistance mechanism unraveled in our study is likely to affect its therapeutic benefit.

In summary, we have reported to our knowledge, a novel function for α2β1 integrin in human effector T cells consisting of mediating MTX resistance. This observation also occurs in effector RA T cells, but because of limited access to RA samples, we could not answer some key questions such as weather α2β1 expression and function correlates with MTX non-responders in RA. Our results do suggest that this could be case. Because α2β1 integrin is expressed on synovial Th17 cells of RA patients (11), it will be interesting to examine whether at the clinical level, it could represent a novel biomarker predicting MTX response in RA.

## Author contributions

AA and MAE contributed to the design and execution of the experiments, data analysis and results interpretation and writing the manuscript. DN performed experiments and analyzed the data. PF designed experiments, analyzed and interpreted the data. FA conceived and designed the study, supervised the work, analyzed the data and wrote the manuscript. All authors read and approved the final manuscript.

### Conflict of interest statement

The authors declare that the research was conducted in the absence of any commercial or financial relationships that could be construed as a potential conflict of interest.

## Abbreviations

ABCC1: ATP Binding Cassette subfamily C Member 1
ABCG2: ATP-binding Cassette subfamily G member 2
Col: collagen
ECM: extracellular matrix
Fbn: fibronectin
Lam: laminin
MFI: mean fluorescence intensity
MTX: methotrexate
NS: non-specific band
PBMCs: Peripheral blood mononuclear cells
PLL: poly-L-lysine
PMA: Phorbol-12-Myristate 13-Acetate
RA: rheumatoid arthritis
sCol: soluble Col
TCR: T cell receptor.
