# Supplementary figures and images for: Alpha2beta1 Integrin (VLA-2) Protects Activated Human Effector T Cells From Methotrexate-Induced Apoptosis
Source: Front Immunol. 2018 Oct 15;9:2269. doi: 10.3389/fimmu.2018.02269 (PMC6197073; doi:10.3389/fimmu.2018.02269)

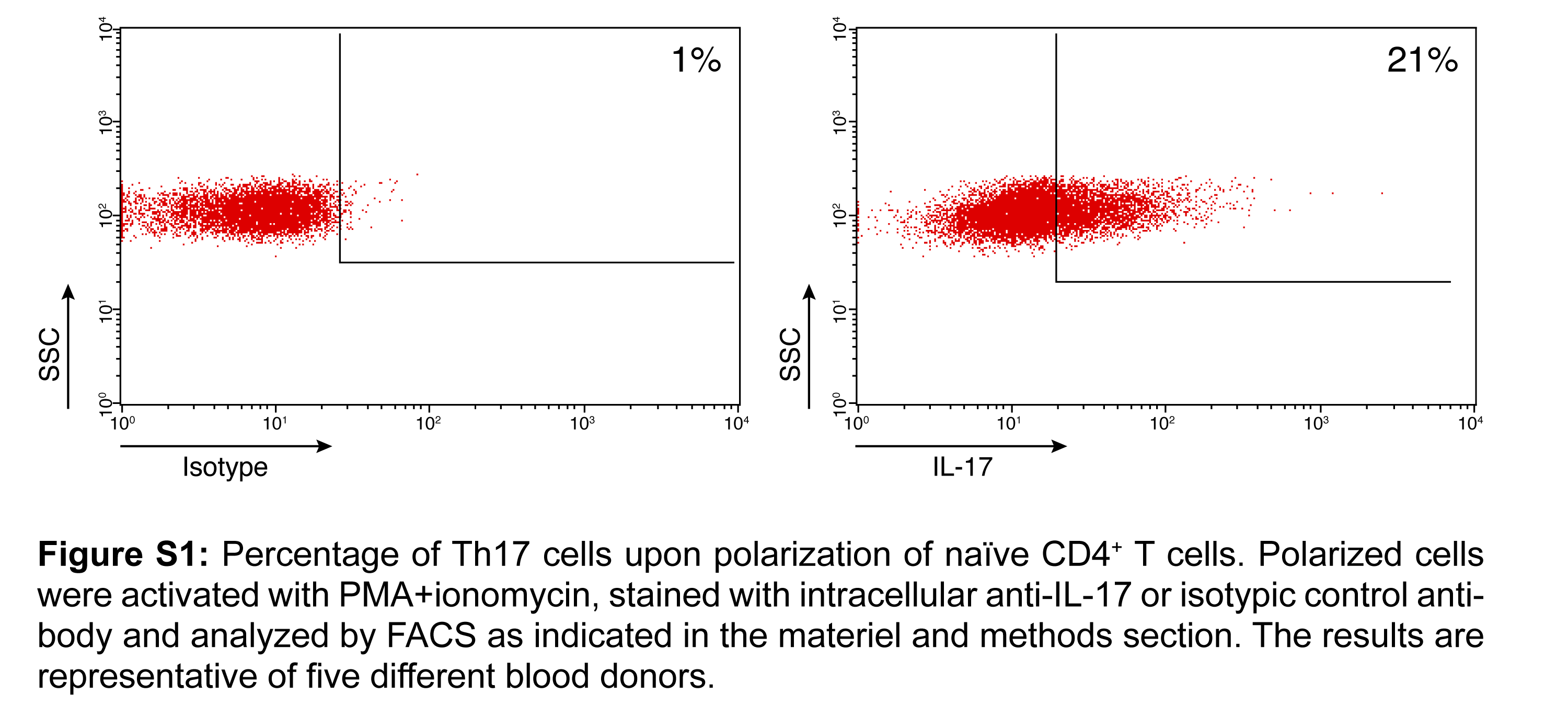

Supplement: Supplementary file 1 [file Image_1.TIF]
